# Magnetic field exposure and long-term survival among children with leukaemia

**DOI:** 10.1038/sj.bjc.6603002

**Published:** 2006-03-21

**Authors:** D E Foliart, B H Pollock, G Mezei, R Iriye, J M Silva, K L Ebi, L Kheifets, M P Link, R Kavet

DE Foliart^*^^,^^1^, BH Pollock^2^, G Mezei^3^, R Iriye^4^, JM Silva^4^, KL Ebi^5^, L Kheifets^6^, MP Link^7^, R Kavet^3^

**Correction to:** *British Journal of Cancer* (2006) **94**, 161–164. doi:10.1038/6602916

Owing to an author error, the legend to Figure 1 was incorrect. The correct legend is given below.

DE Foliart, BH Pollock, G Mezei, R Iriye, JM Silva, KL Ebi, L Kheifets, MP Link, R Kavet

## Figures and Tables

**Figure 1:**
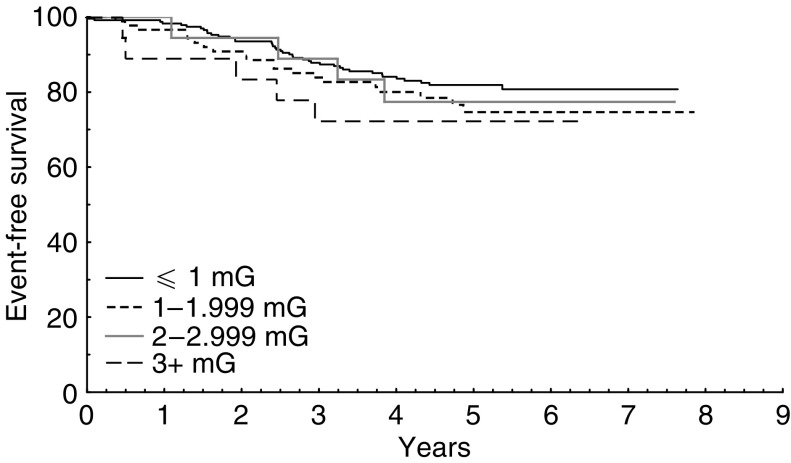
Kaplan–Meier estimates for event-free survival among children with B-precursor ALL, stratified by 24-h TWA MF exposure (log rank test *P*=0.54).

